# Temporal Proteomic Profiling of Hospitalized COVID-19 Patients Reveals Prognostic Biomarkers

**DOI:** 10.1021/acs.jproteome.6c00129

**Published:** 2026-07-29

**Authors:** Mauricio Quiñones-Vega, Patricia Sosa-Acosta, Giovanna Câmara Giudicelli, Mariléa Furtado Feira, Bibiana S. O. Fam, Nathan Araújo Cadore, Renan Cesar Sbruzzi, Fernanda Sales Luiz Vianna, Fábio C. S. Nogueira, Gilberto B. Domont

**Affiliations:** † Proteomics Unit, Institute of Chemistry, Federal University of Rio de Janeiro (UFRJ), Rio de Janeiro 21941-901, Brazil; ‡ Laboratory of Proteomics (LabProt), LADETEC, Institute of Chemistry, Federal University of Rio de Janeiro (UFRJ), Rio de Janeiro 21941-901, Brazil; § Precision Medicine Research Center, Institute of Biophysics Carlos Chagas Filho, Federal University of Rio de Janeiro (UFRJ), Rio de Janeiro 21941-901, Brazil; ∥ Laboratory of Immunogenetics, Department of Genetics, Federal University of Rio Grande do Sul (UFRGS), Porto Alegre 90035-903, Brazil; ⊥ Genomic Medicine Laboratory, Experimental Research Center, Hospital de Clínicas de Porto Alegre (HCPA), Porto Alegre 90035-903, Brazil

**Keywords:** COVID-19, SARS-CoV-2 infection, plasma proteomics, biomarker, survival prediction

## Abstract

COVID-19 presents a wide spectrum of clinical outcomes, and early identification of patients at high-risk for a worse disease prognosis remains a critical challenge. In this study, we performed a six-week longitudinal proteomic analysis of 197 plasma samples collected weekly from 44 hospitalized COVID-19 patients. Temporal profiling revealed 828 proteins with significant time-dependent abundance changes, clustering into an early acute-phase and inflammatory response group that gradually declined toward the cohort’s overall mean profile over time and a group of late vesicle-mediated transport and antigen binding that were decreased in the first weeks. Global correlation mapping identified six coregulated protein modules, notably including insulin-like growth factor binding proteins. Proteomic divergence between survivors and nonsurvivors became pronounced from week 4 onward. The proteins HPR, IGFALS, PF4, LPA, and COLEC11 achieved AUCs > 0.72 for mortality prediction in the first 2 weeks, and an SVM model combining these markers yielded an AUC of 0.801. These findings demonstrate that plasma proteome trajectories capture dynamic host responses and generate candidate prognostic markers, offering a promising strategy for risk stratification and guiding targeted interventions in COVID-19.

## Graphical abstract

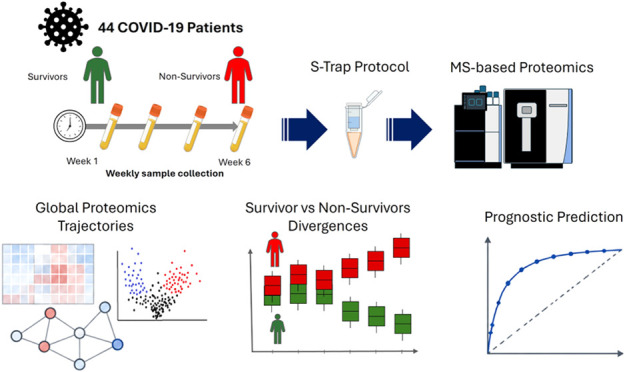

## Introduction

COVID-19, the disease caused by the SARS-CoV-2 coronavirus, presents a wide range of clinical manifestations, varying from asymptomatic cases to severe outcomes, including death. Although most patients exhibit mild symptoms such as fever, dry cough, and fatigue, a study conducted on a Chinese cohort of 1099 unvaccinated patients reported that approximately 19% of infected individuals may develop more severe symptoms. Moreover, patients can rapidly progress to critical conditions if medical treatment is not adequate.^1^ Therefore, it is essential not only to control the disease, but also to monitor its progression to identify the factors that determine whether a patient recovers or deteriorates. This enhances our understanding of SARS-CoV-2 pathophysiology but also aids in identifying biomarkers that define disease severity, have prognostic value, or indicate a specific stage of the illness. Additionally, monitoring the infection allows us to determine which medical treatments are truly effective in improving patient health, a crucial aspect for appropriate clinical management and identifying therapeutic targets, paving the way for developing new treatment strategies to combat COVID-19.

Alterations in human plasma proteins are recognized as indicators of physiopathological changes caused by various diseases, including viral infections. In this regard, mass spectrometry (MS)-based proteomics has become a fundamental tool, enabling the characterization of proteins in a sample. Despite the challenge posed by the wide dynamic range of plasma protein concentration, the continuous improvement of proteomics in sensitivity, robustness, and higher throughput has led to its application in large-scale clinical studies for the systemic characterization of disease responses.^2^ Specifically, for COVID-19, several groups have analyzed the serum or plasma proteome of infected patients in large-scale clinical studies. Zhong^3^ et al. by monitoring the plasma protein profile of 50 COVID-19 patients in samples collected within 24 h after confirmation of the infection and again after 14 days, identified 200 proteins with significantly altered abundance, some of them related to cytokine response and other immune system functions. Imatinib treatment also attenuated plasma proteomic alterations caused by endothelial dysfunction and alveolar damage.^4^ Demichev^5^ et al. characterized the temporal progression of COVID-19 in 139 patients by analyzing 687 plasma samples, showing it is possible to predict patient trajectories based on the initial response through the identification of proteomic and diagnostic markers indicating the need for mechanical ventilation. Our study aims to characterize the trajectory of COVID-19 in the plasma of unvaccinated Brazilian patients using a proteomic approach combined with clinical and diagnostic parameters, to understand the comprehensive molecular framework that highlights alterations in different disease phenotypes, depending on severity, age, or progression.

## Experimental Procedures

### Study Cohort

Plasma samples from 44 COVID-19 patients were supplied by the Biobank of Hospital de Clínicas de Porto Alegre (10.22491/hcpa-biobanco-amostras). They were collected weekly from the hospitalized patients (Table S1), covering a period of up to 6 weeks from the first day of sampling. All patients tested positive for SARS-CoV-2 by RT-PCR and were unvaccinated at the time of hospitalization. Sample collection occurred from March 2020 through April 2021. Nine patients had samples collected in all 6 weeks, 12 in at least 5 weeks, 16 in 4 weeks, five in 3 weeks, and two in 2 weeks, totaling 197 samples collected in general wards and Intensive Care Units. Clinical and clinical chemistry data of patients were retrieved from medical records (Table S2).

### Sample Processing for Proteomic Analysis

We processed the plasma samples using the S-trap protocol as described by Zougman^6^ et al. Briefly, we diluted 1.6 μL of plasma (corresponding to ∼100 μg of protein, considering ∼60 mg/mL of protein concentration in plasma) 20-fold with 5% SDS in 50 mM TEAB at pH 8.5. Reduction was carried out using DTT for 1 h at 30 °C, followed by alkylation with 40 mM IAA for 45 min at room temperature, protected from light. After acidifying the mixture with phosphoric acid to reach a final concentration of 1.2%, we added 350 μL of 90% methanol in 100 mM TEAB (Binding Buffer). The samples were then transferred to S-Trap mini spin columns (Protifi, USA) and centrifuged at 4000*g* for 30 s. To remove the detergent, we washed the columns three times with 450 μL of binding buffer. For digestion, we used trypsin (Promega) at a 1:50 ratio (μg of trypsin to μg of protein) in 125 μL of 50 mM TEAB at pH 8.5 and incubated the samples for 18 h. Peptides were eluted sequentially with four solutions: 80 μL of 50 mM TEAB (pH 8.5), 80 μL of 0.1% formic acid (FA), 40 μL of 50% acetonitrile (ACN)/0.1% FA, and 40 μL of 70% ACN/0.1% FA. Each step was followed by centrifugation at 4000*g* for 60 s. Finally, we dried the samples using a speed-vac (SAVANT, Thermo Fisher Scientific) and stored them at −30 °C until mass spectrometry analysis.

### Mass Spectrometry Acquisition and Data Analysis

Tryptic peptide mixtures were resuspended in 0.1% formic acid and quantified using the Qubit fluorometric assay following the manufacturer’s instructions. From each sample, we applied 2 μg of peptides into a Vanquish Neo UHPLC System (Thermo Fisher Scientific) coupled to an Orbitrap Exploris 480 mass spectrometer (Thermo Fisher Scientific). Peptides were loaded onto a PepMap Neo Trap Cartridge precolumn, 5 mm (length) × 300 μm (internal diameter), with a 5 μm particle size, then fractionated onto an EASY-Spray column, 50 cm × 75 μm ID, PepMap RSLC C18, 2 μm (Thermo Fisher Scientific). For mobile phases, we use 0.1% formic acid (solvent A) and 80% ACN/0.1% formic acid (solvent B). The peptide mixture was separated with a gradient of 2–23% B for 70 min, 23–45% B for 10 min, 45–99% B for 0.1 min, and 99% B for 10 min at a flow rate of 300 nL/min and a column temperature of 60 °C. The data-independent acquisition (DIA) method consisted of a full MS scan from 375–1500 m/z with a resolution of 30,000 at m/z 200, to several MS2 windows with variable isolation widths and an NCE of 28. The acquisition window covered an m/z range from 375 to 1498 through 59 consecutive isolation windows with 0.5 m/z margins. The first 11 windows had a width of 16 m/z, followed by 24 with 10 m/z, 7 with 15 m/z, 6 with 20 m/z, 9 with 40 m/z, and finally, 2 windows with 91 m/z. The target AGC was set to 1e6 with a maximum automatic injection time.

### Proteomic Data Processing and Analysis

We imported the raw files into DIA-NN 1.8.1 ^7^ in the LC robust (high precision) mode using median run-based normalization dependent retention time (TR). Mass tolerances for MS2 and MS1 were set to automatic. For identification, we used a spectral library previously created from DDA runs of plasma, containing 1927 protein groups and 53,207 precursors. Additional parameters were defined as follows: peptide length range, 7–30 aa; precursor charge range, 2–4; precursor m/z range, 300–1500; fragment ion m/z range, 200–1800; static modification, carbamidomethylation of cysteine; and N-terminal methionine excision; also, a maximum of 2 variable modifications were defined, including oxidation of methionine and N-terminal acetylation. For protein inference, the FDR was adjusted to 1% at the precursor, peptide, and protein levels.

Protein abundance values were first log_2_-transformed and normalized by subtracting the median intensity of each sample (Table S3). After this transformation, potential contaminants were removed. Proteins only quantified in at least 70% of all samples were retained. Missing values were then imputed in Perseus (version 1.6.15.0) using a normal distribution approach (width = 0.3, down shift = 1.8). We then performed a one-sample *t* test at each week comparing protein abundances to the cohort mean profile, applying Benjamini–Hochberg multiple testing correction with a false discovery rate (FDR) threshold of 0.05 (Table S4). The hierarchical clustering analysis was done using the Z-scored intensities of all significantly altered proteins at each time point. Pearson Correlation was used as distance metric and average linkage as agglomeration method. Differential abundance between survivors and nonsurvivors at each time point was assessed using two-sample Student’s *t* tests. Multiple-testing correction using the Benjamini–Hochberg FDR procedure was evaluated; however, due to the limited statistical power and high interindividual variability, no proteins passed the FDR threshold. We therefore report raw *p*-values and corresponding log_2_ fold-changes for these tests (Table S5). Gene Ontology (GO) enrichment analysis was performed using ClusterProfiler R package v4.6.2,^8^ using all human proteins annotated in org.Hs.eg.db, *p*-values were computed by the hypergeometric (Fisher) test and adjusted for multiple testing using the Benjamini–Hochberg procedure. To show enriched ontology clusters across weeks we use the bioinformatics resource Metascape^9^ (https://metascape.org/) applying default settings. Metascape performs groups related terms into clusters based on kappa-statistical similarity, selecting the most significant term from each cluster for heatmap visualization. Longitudinal changes in protein abundance were additionally evaluated using a linear mixed-effects model, with patient identity included as a random effect to account for repeated measures and time and clinical outcome modeled as fixed effects; significance was assessed using Benjamini–Hochberg FDR correction (FDR < 0.05).

ROC analyses of individual proteins were performed using the Metaboanalyst platform (https://www.metaboanalyst.ca/). To evaluate the prognostic value of a custom panel of five proteins: HPR (Uniprot accession: P00739), IGFALS (Uniprot accession: P3585), LPA (Uniprot accession: P08519), PF4 (Uniprot accession: P02776), COLEC11 (Uniprot accession: Q9BWP8), we trained a linear support vector machine (SVM) using a leave-one-patient-out (LOPO) strategy. Both training and test sets were restricted to samples collected in week 1 or week 2. In each iteration, all samples from one patient were held out as the test set, and the model was trained exclusively on samples from the remaining patients. Preprocessing steps were computed using only the training data and then applied to the held-out patient. Receiver operating characteristic (ROC) analysis and confidence intervals were computed at the patient level using DeLong’s method. We computed the standard diagnostic metrics, including sensitivity, specificity, positive and negative predictive values, and overall accuracy based on true positives, false positives, true negatives, and false negatives values obtained by applying a fixed probability threshold (0.5) to classify each sample as death or survival.

## Results

In the present study, 44 patients were included (30 men and 14 women); 21 died, 18 were discharged, and 5 transferred to another hospital without information on the outcome. To monitor the trajectory of these patients during their hospitalization with COVID-19, plasma was collected weekly for 6 consecutive weeks, totaling 197 samples. The collected samples were processed using the S-Trap protocol, and data were acquired in Data-Independent Acquisition (DIA) mode following a label-free quantification strategy (Figure 1A). A total of 1525 proteins were identified after contaminant removal (Table S3), averaging 1044 proteins per sample.

**Figure 1:**
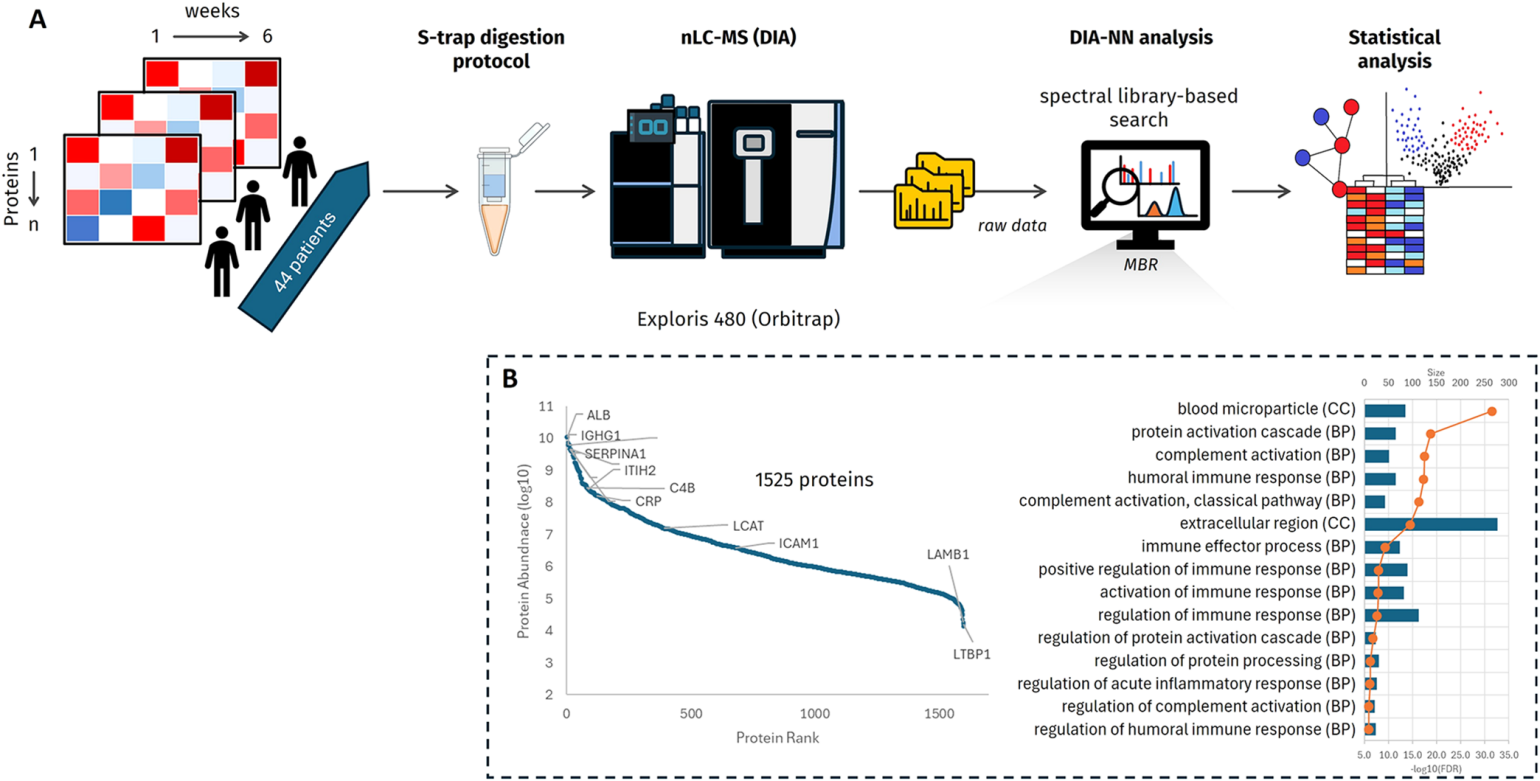
(A) Schematic for the proteomic analysis of sequentially collected samples from 44 COVID-19 patients. (B) Abundance values of 1525 plasma proteins detected in the study. (C) The 15 most significant terms obtained from 1D enrichment of all detected proteins.

After additionally excluding proteins not quantified in at least 70% of samples, 851 proteins remained for quantitative analysis. The most abundant protein detected was albumin (ALB), proteins like CRP, apolipoproteins, and α-1-antitrypsin, previously associated with COVID-19,^10–12^ were also identified, and LTBP1 was the least abundant protein. The most significant biological annotations were related to blood microparticles, protein activation cascade, complement activation, and humoral response, which are common processes in response to COVID-19 (Figure 1B).

### Changes in the Plasma Proteome During COVID-19 Progression

Our primary objective was to investigate the dynamic changes in the plasma proteome during SARS-CoV-2 infection. Since plasma reflects the physiological state of the patient, protein abundance is expected to fluctuate over time and according to disease severity. To examine the proteins that change in each time frame relative to the global profile, we perform a one-sample *t* test for each week. This analysis helps identify proteomic profiles that may reflect the transition from the acute to the chronic phase of the disease

Throughout the patients’ trajectories, 828 proteins exhibited statistically significant changes in abundance relative to the mean abundance at each time point (Table S4). The hierarchical clustering analysis stratified proteins into four groups based on their temporal response during COVID-19 progression. Nearly 50% of the significantly altered proteins exhibited increased abundance in the first week of hospitalization, followed by a decline throughout the disease (Group 1, 237 proteins; Figure 2A). The enrichment of functional annotations in this group is associated with early responses to COVID-19, as the acute phase response, the inflammatory response, and with the Hp-Hb complex formed from the hemolysis of erythrocytes. Proteins in Group 2 increased between weeks 1 and 2, stabilizing from week 3 until week 6 (Group 2, 340 proteins; Figure 2A). The most significant annotations in Group 2 include antigen binding, endocytosis, and vesicle-mediated transport. The remaining proteins displayed heterogeneous behavior, with varying levels of increase or decrease at different time points. Next, we investigated the global regulation of the plasma proteome to identify protein networks that are being coregulated during COVID-19. To achieve this, we performed an unsupervised hierarchical clustering of correlation coefficients between proteins (Figure 3A, 362,100 correlation coefficients). In this study, we considered up to 197 abundance values for each protein and included the 851 proteins quantified in at least 70% of the samples.

**Figure 2:**
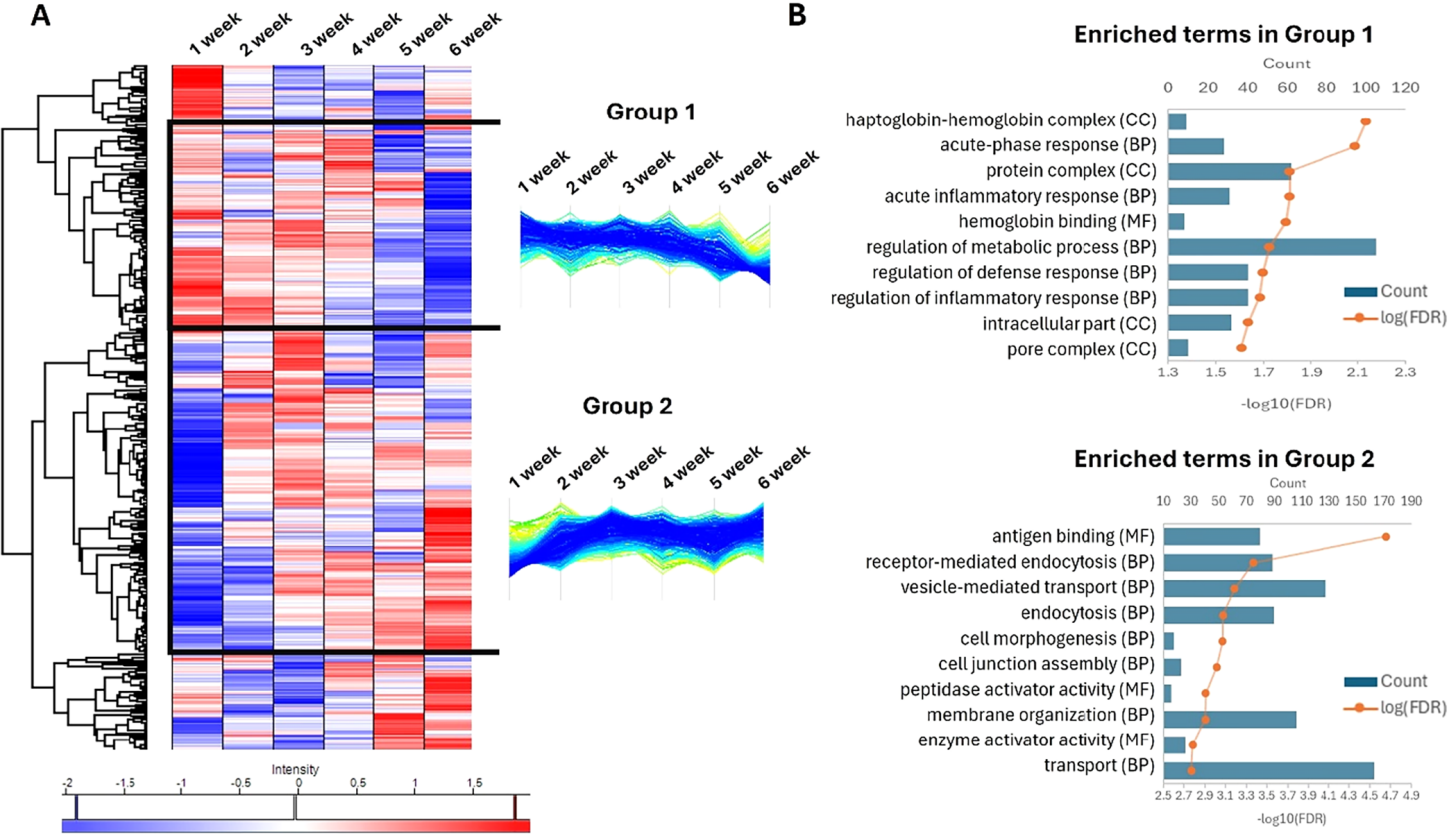
Longitudinal Trajectories and Functional Interpretation of Plasma Proteins Significantly Altered During SARS-CoV-2 Infection. (A) Hierarchical clustering of plasma proteins significantly altered across the six analyzed time points. Two major groups exhibit time-dependent trends: proteins in Group 1 show a decreasing trend over time, whereas proteins in Group 2 increase in abundance, particularly during the first 2 weeks of infection. (B) Fisher’s exact test was used to identify the most represented biological processes (BP), molecular functions (MF), and cellular components (CC) in each group.

**Figure 3:**
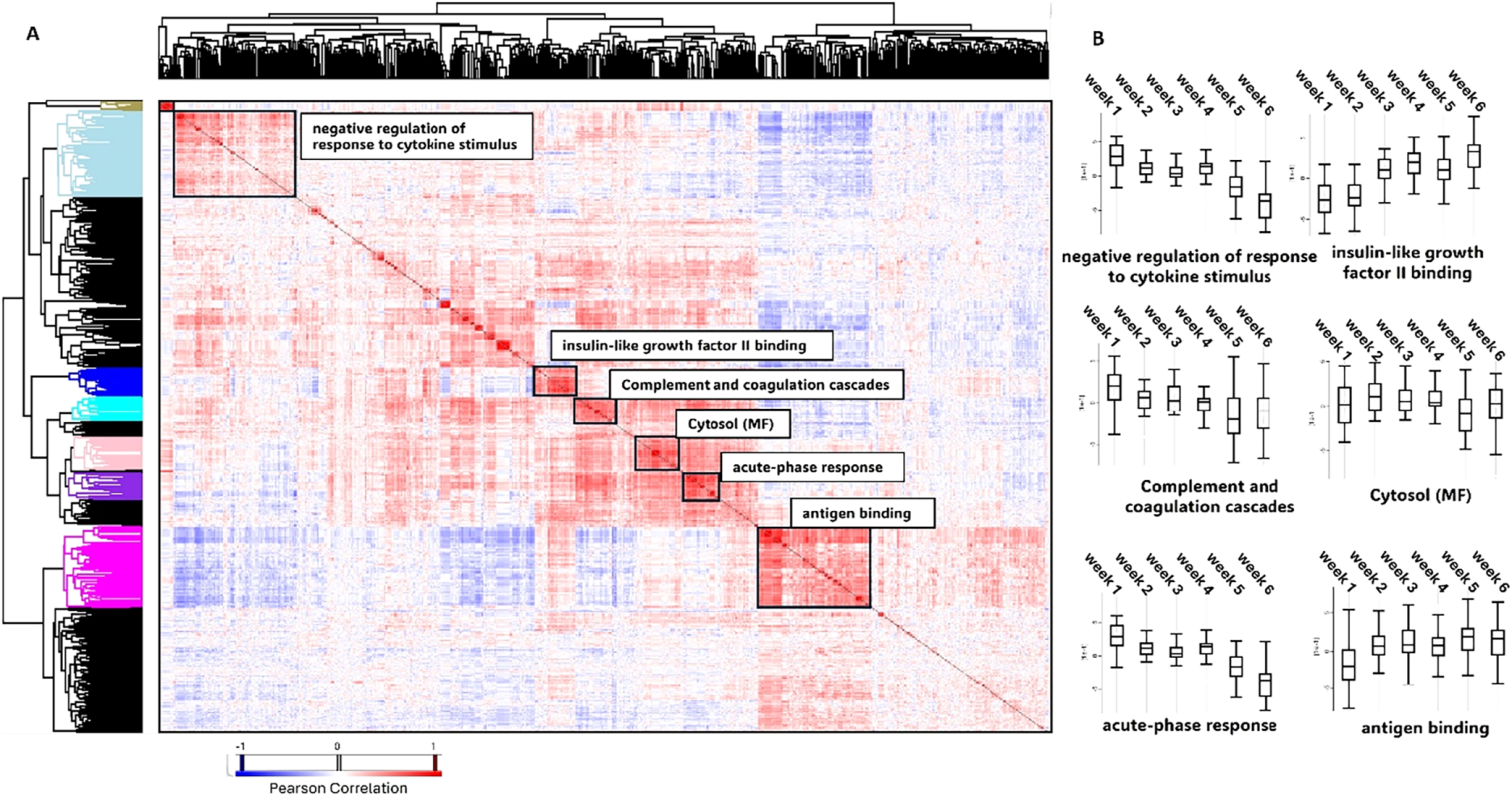
Global Correlation Map of the Plasma Proteome. (A) Pairwise protein–protein correlation matrix computed using log2-transformed intensity of all 851 proteins. Each cell represents the Pearson correlation coefficient between a pair of proteins across all available samples; The color scale indicate the strength and direction of the correlation, from blue (strong negative correlation) through white (no correlation) to red (strong positive correlation). (B) Boxplot representing the normalized abundances of proteins from different clusters observed in the global correlation map over the 6 weeks of patient hospitalization.

Six distinct groups of correlated proteins were identified (Figure 3A,B). The most representative term in the first group, which included 118 proteins, was associated with the response to cytokine stimuli, as expected, given that SARS-CoV-2 triggers hyperactivation of the inflammatory response, leading to the well-documented cytokine storm. In addition, a group of 36 proteins related to the acute phase of immune response were identified, including CRP, SAA1, SAA2, ITIH3, ITIH4, SERPINA3, and SERPINA4, all of which are linked to infectious processes.^13,14^ Other highlighted groups included proteins involved in the complement cascade and coagulation (35 proteins), antigen-binding proteins (109 proteins), that included multiple immunoglobulins, and 44 intracellular proteins, probably originating from tissue leakage. Interestingly, a cluster of proteins associated with insulin-like growth factor (IGF) binding showed an increasing trend during the disease (Figure 3B).

### Comparison between Survivors and Non-Survivors

To identify potential prognostic protein markers that could predict patient outcomes from the early stages of the disease, we analyzed changes in the plasma proteome by grouping samples based on patient outcomes (survivors or nonsurvivors). To assess plasma dynamics in these patients, we performed Principal Component Analysis (PCA) for each collection time point (Figure 4A and Table S6). Although no notable differences were observed between these two groups of patients in the early stages, from the fourth week onward the proteomic profile tends to separate better. To determine the proteins responsible for this divergence in the proteome, we conducted Student’s *t* tests at each time point, comparing deceased patients to those who were discharged (Table S5). This analysis identified 41, 80, 81, 180, 153, and 107 differentially expressed proteins in the first through sixth weeks, respectively (Figure 4B).

**Figure 4:**
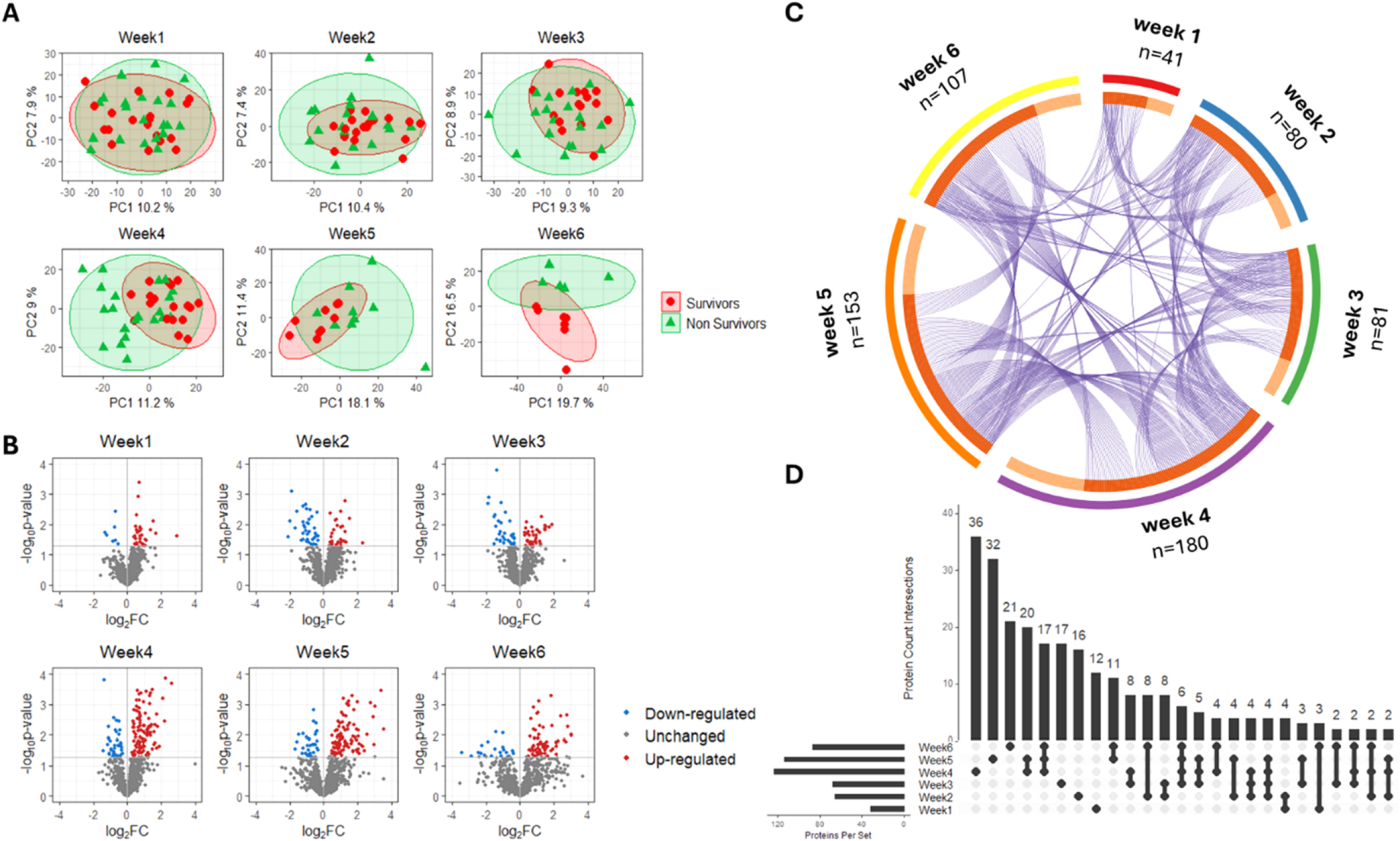
Comparison Between Deceased and Discharged Patients at Each Time Point. (A) Principal Component Analysis (PCA) of the deceased (green) and discharged (red) patient groups for each week. The ellipsoids represent the bivariate normal distribution around the data points. (B) Volcano plot showing upregulated (red) and downregulated (blue) proteins in deceased patients compared to survivors at each analyzed time point. (C) Circle plot illustrating the overlap of differentially expressed proteins across the weeks. (D) Upset plot representing the intersections of differentially expressed proteins across different time points.

Despite no notable difference in global proteome of the groups in the first 3 weeks of hospitalization, 122 proteins were found differently abundant at these time points. Also, most of the differentially expressed proteins observed were found in common in more than 1 week. Figure 4C illustrates the overlap of significant proteins across the six groups. The fourth and fifth weeks showed the highest numbers of unique proteins, with 36 and 32 identified, respectively. This was followed by 21 unique proteins detected in the sixth week. The third week yielded 17 unique proteins, while the first and second weeks showed the lowest counts, with 12 and 11 unique proteins, respectively (Figure 4D). Only IGFALS was found to be significantly altered across all time points, while 20 differentially expressed proteins were detected in at least four and 10 of these proteins where also found significant in the mixed effect model, including IGFALS and PF4 (Table 1).

**Table 1:** Significant Proteins Differentiating Survivors and Non-Survivors Identified by Student’s t-test and Consistently Detected in at Least 4 Weeks

*Gene*	uniprot ID	protein name	Count*	adj.*p*-value outcome × time (interaction)
*IGFALS*	P35858	Insulin-like growth factor-binding protein complex acid labile subunit	6	0,038
*HPR*	P00739	Haptoglobin-related protein	5	0,308
*IGFBP7*	Q16270	Insulin-like growth factor-binding protein 7	5	0,105
*LGALS3BP*	Q08380	Galectin-3-binding protein	4	0,178
*PF4*	P02776	Platelet factor	4	0,004
*PROCR*	Q9UNN8	Endothelial protein C	4	0,389
*IGFBP3*	P17936	Insulin-like growth factor-binding protein 3	4	0,065
*EFEMP1*	Q12805	EGF-containing fibulin-like extracellular matrix protein 1	4	0,146
*ICAM1*	P05362	Intercellular adhesion molecule 1	4	0,011
*CRYZ*	Q08257	Quinone oxidoreductase (EC 1.6.5.5)	4	0,001
*LCN2*	P80188	Neutrophil gelatinase-associated lipocalin	4	0,004
*LPA*	P08519	Apolipoprotein(a) (EC 3.4.21.-)	4	0,568
*HERC4*	Q5GLZ8	Probable E3 ubiquitin-protein ligase HERC4	4	0,153
*TSKU*	Q8WUA8	Tsukushi (E2-induced gene 4 protein)	4	0,006
*LRRC8A*	Q8IWT6	Volume-regulated anion channel subunit LRRC8A	4	0,009
*COLEC11*	Q9BWP8	Collectin-11	4	0,308
*LYVE1*	Q9Y5Y7	Lymphatic vessel endothelial hyaluronic acid receptor 1	4	0,039
*APOL1*	O14791	Apolipoprotein L1	4	0,002
*CALR*	P27797	Calreticulin	4	0,056
*CD163*	Q86VB7	Scavenger receptor cysteine-rich type 1 protein M130	4	0,01

In the first 2 weeks, the main differentially altered biological processes were related to complement activation and opsonization. Additionally, humoral response and other terms such as granulocyte and leukocyte migration were decreased or did not change in nonsurvivors (z-score ≤ 0). From the fourth week onward, there was an increase in the acute-phase inflammatory response in deceased individuals; processes such as leukocyte migration, previously decreased, became upregulated, and blood coagulation and complement activation continued upregulated (Figure 5A and Table S7).

**Figure 5:**
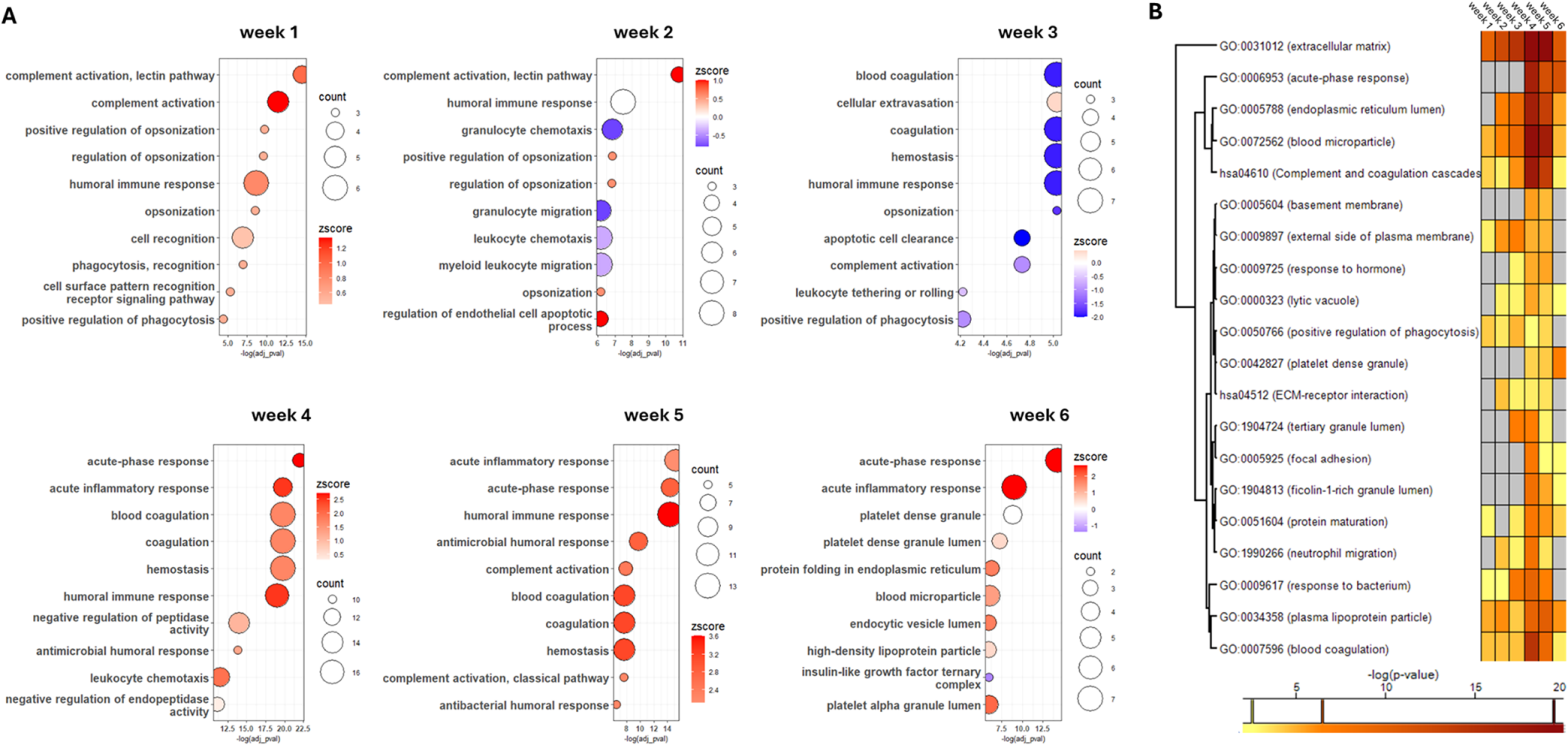
Functional analyses of differentially expressed proteins between nonsurvivors and survivors across the 6 weeks of hospitalization. (A) For each week, the 10 most significant biological terms identified by over-representation analysis are shown. Weekly lists of significant proteins (Benjamini–Hochberg FDR < 0.05) were analyzed using the enrichGO function from the ClusterProfiler package against the human annotation database org.Hs.eg.db. Circle size reflects the number of proteins associated with each term. Bubble colors represent the z-score, that summarizes the direction of change of the proteins in each term (positive z-score = higher in nonsurvivors; negative z-score = higher in survivors). (B) Heatmap showing enrichment patterns of differentially expressed proteins lists across the 6 weeks, with color intensity corresponding to the negative log* p*-value; gray cells indicate that the term was not significantly enriched in that week.

The data were also analyzed using the Metascape portal (Figure 5B and Table S7). The blood coagulation process was significant at all time points, indicating its alteration over time, with the highest representation in weeks 4 and 5 of hospitalization. Annotations such as acute phase, neutrophil migration, and plasma lipoprotein particles which are typically associated with inflammation, appeared more prominently from week 3 onward, consistent with previous observations.

To account for the repeated-measures structure of the data set, we complemented the time-specific Student’s *t* tests with a linear mixed-effects model. This model included patient as a random intercept to account for within-patient correlation, and time (week), outcome (survivor vs nonsurvivor), and their interaction as fixed effects. These models were used to confirm whether proteins identified in the week-by-week *t* tests also showed statistically robust longitudinal differences after accounting for repeated measurements and uneven follow-up.

Across all quantified proteins, the mixed-effects model indicated that survivors and nonsurvivors did not differ in their overall proteome abundance, especially in the first 3 weeks, as no proteins showed a significant main effect of outcome. However, 123 proteins displayed a significant time-by-outcome interaction (Figure 6A). These proteins segregated into two distinct temporal clusters (Figure 6B). The first cluster comprised proteins whose abundance increased over time in nonsurvivors relative to survivors, whereas the second cluster exhibited the opposite pattern. Functional enrichment analysis revealed that proteins in Cluster 1 were predominantly associated with regulation of proteolysis, leukocyte migration, and phagocytosis, while proteins in Cluster 2 were enriched for pathways related to wound healing, humoral immune responses, and acute inflammatory processes (Figure 6C).

**Figure 6:**
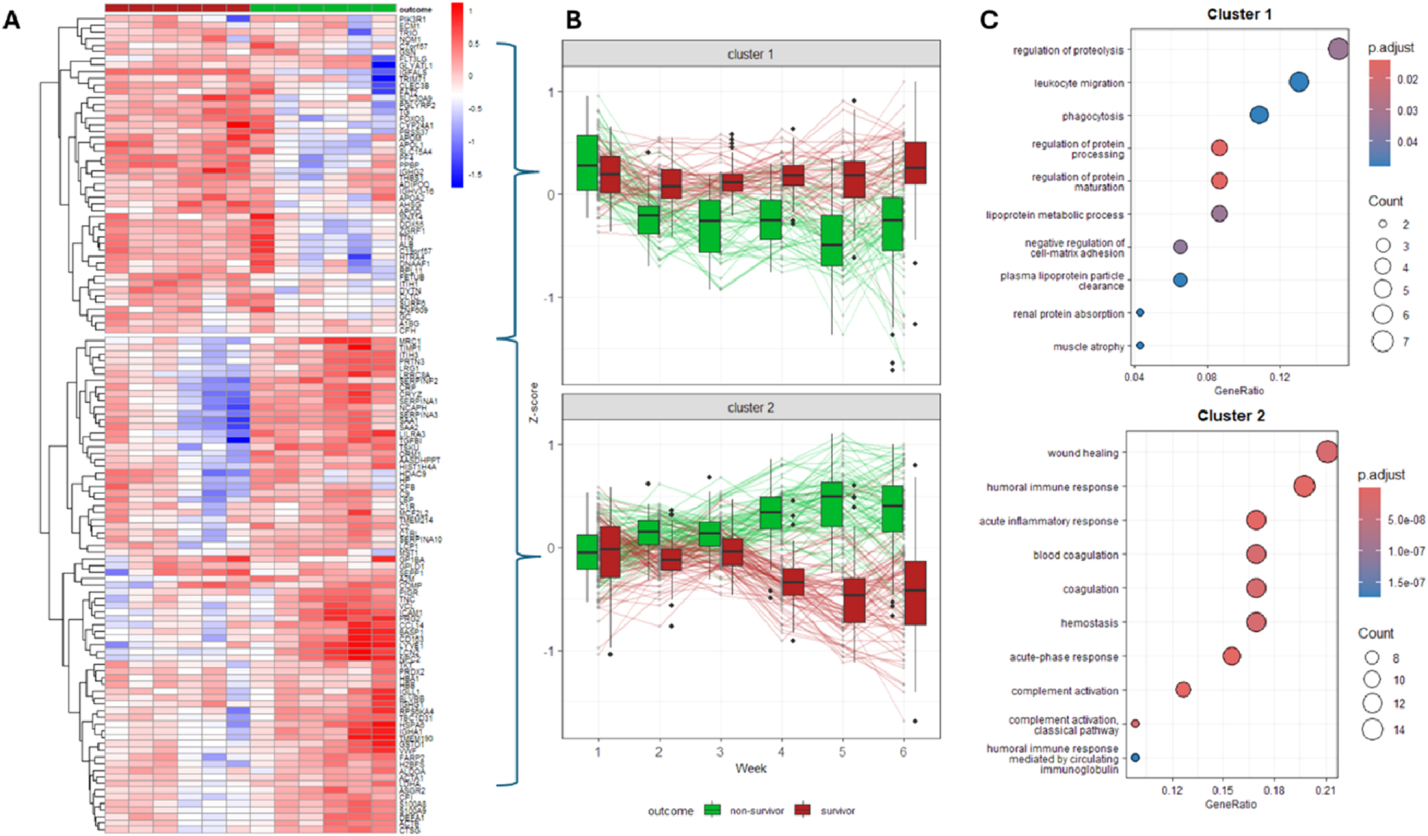
Longitudinal analysis of proteins exhibiting a significant time-by-outcome interaction according to mixed-effect model. (A) Hierarchical clustering of significant proteins revealing two major expression clusters distinguishing survivors from nonsurvivors over the six-week period. (B) Cluster-specific temporal trajectories showed that proteins in Cluster 1 increased in abundance over time in nonsurvivors relative to survivors, whereas proteins in Cluster 2 displayed the opposite pattern. (C) the 10 most represented Gene Ontology term enriched in the two clusters.

### Prediction of Survival or Death (ROC Curve for Biomarkers)

To determine which proteins to use to predict patient survival from the early stages, we focused on samples from the first 2 weeks of hospitalization, as the PCA evaluation (Figure 6A) showed minimal separation in plasma profiles at these time points. We also assessed the performance of each protein individually as a survival biomarker using a classical ROC curve analysis. The ROC curve summarizes the sensitivity and specificity of a given molecule in accurately classifying the two condition groups. Table 2 presents the 35 proteins with an area under the curve (AUC) greater than 0.70. Among them, the proteins HPR (AUC 0.805), IGFALS (AUC 0.795), PF4 (AUC 0.755), LPA (AUC 0.758), and COLEC11 (AUC 0.723) were significantly different between deceased and surviving patients in at least four time points, as determined by the *t* test (Table 1).

**Table 2:** Classical ROC Curve Analysis for Proteins with an Area under the Curve (AUC) > 0.70

uniprot ID	genes	AUC	*p*-value	fold-change
**P00739**	*HPR*	0.805	9.57 × 10^–05^	–0.92409
**P35858**	*IGFALS*	0.795	0.0003	–0.90394
**Q92569**	*PIK3R3*	0.774	0.006	0.76458
**A0A0C4DH36**	*IGHV3–38*	0.758	0.002	0.87149
**P08519**	*LPA*	0.758	0.002	–0.8365
**Q5T6F0**	*DCAF12*	0.757	0.008	0.75065
**P02776**	*PF4*	0.755	0.001	–0.93937
**Q68DL7**	*C18orf63*	0.750	0.000	1.0124
**P10720**	*PF4 V1*	0.748	0.003	–0.83455
**A0A075B6H9**	*IGLV4–69*	0.746	0.001	–0.88072
**Q9H4L5**	*OSBPL3*	0.746	0.023	0.65194
**P02775**	*PPBP*	0.737	0.002	–0.90202
**Q6EMK4**	*VASN*	0.737	0.008	0.73361
**Q9H813–2**	*PACC1*	0.737	0.001	0.92839
**P13598**	*ICAM2*	0.734	0.005	0.82008
**P07996**	*THBS1*	0.730	0.008	–0.77291
**P12111**	*COL6A3*	0.730	0.003	0.92999
**P15144**	*ANPEP*	0.730	0.003	–0.85479
**Q9UGM5**	*FETUB*	0.727	0.060	–0.58403
**P01743**	*IGHV1–46*	0.725	0.008	0.77579
**Q9BWP8**	*COLEC11*	0.723	0.010	0.74585
**P33151**	*CDH5*	0.722	0.003	0.8659
**Q99538**	*LGMN*	0.718	0.004	0.84135
**P01023**	*A2M*	0.717	0.004	0.84763
**Q6P4Q7**	*CNNM4*	0.717	0.007	–0.70691
**O14733**	*MAP2K7*	0.711	0.018	0.63199
**P10124**	*SRGN*	0.711	0.009	–0.73053
**P04075**	*ALDOA*	0.708	0.009	–0.79996
**O75022**	*LILRB3*	0.706	0.057	0.41712
**Q8N6C8**	*LILRA3*	0.706	0.040	0.4612
**P09486**	*SPARC*	0.704	0.004	–0.80255
**O75019**	*LILRA1*	0.703	0.239	0.32246
**Q9Y5H5**	*PCDHA9*	0.703	0.190	0.40126
**Q15485**	*FCN2*	0.703	0.009	–0.75657
**A0A075B6J9**	*IGLV2–18*	0.701	0.012	0.77681

We then considered this panel of five significant proteins with an AUC greater than 0.7 to develop a combined predictive model using the Support Vector Machine (SVM) algorithm. For model training, we provided 23 samples from surviving patients (12 from the first week and 11 from the second week) and 25 from nonsurviving patients (13 from the first week and 12 from the second week). This training set aimed to optimize the model’s ability to distinguish between survival outcomes based on the proteomic profile.

The ROC analysis based on the five selected proteins yielded an AUC of 0.801 (95% CI: 0.622–0.98), indicating good discriminative ability for survival classification (Figure 7C). To further assess model performance across disease progression, we evaluated 8 unlabeled samples, including early (week 1, *n* = 4; week 2, *n* = 4) and late (week 6, *n* = 12) time points, with balanced group representation (Table 3). Based on these results, we calculated the probability of a patient not surviving if classified as “nonsurvivor” by the model, known as the positive predictive value (PPV), and the probability of a patient being discharged when classified as “discharged“ by the model, known as the negative predictive value (NPV). Importantly, predictive performance metrics were calculated using only early stage samples (weeks 1–2), consistent with the training design of the model and later time points were used as an exploratory robustness check and were not included in the validated performance metrics. Under this constraint, the model achieved a positive predictive value (PPV) of 100% and a negative predictive value (NPV) of 66.7%, with a sensitivity of 50% and specificity of 100%, resulting in an overall accuracy of 75%.

**Figure 7:**
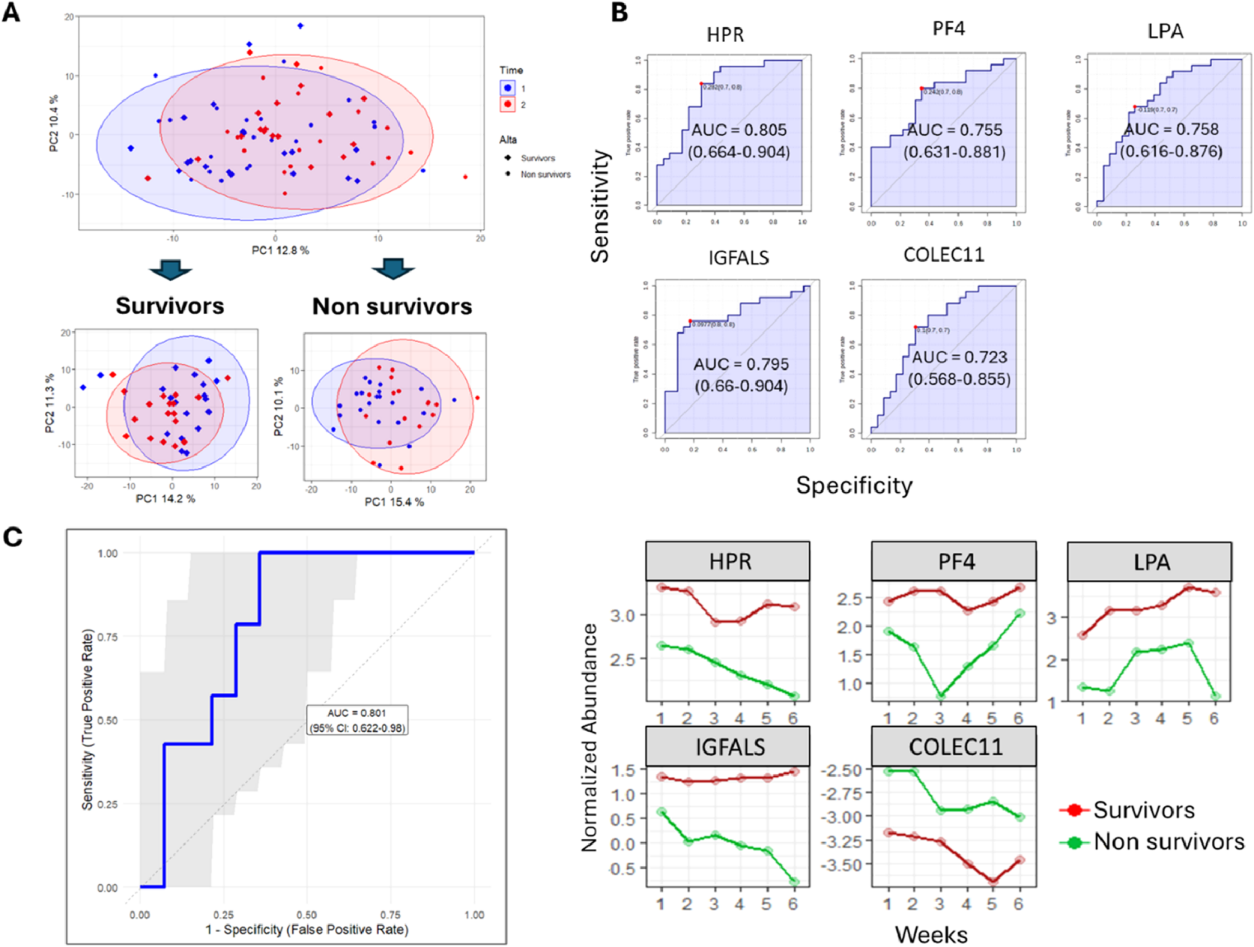
Biomarker analysis based on plasma trajectories of SARS-CoV-2-infected patients. (A) Principal Component Analysis (PCA) of plasma samples collected during the first 2 weeks (blue represents first-week samples, red represents second-week samples). The lower PCAs display the separation of samples by patient outcome: survivors (left) and nonsurvivors (right). (B) Receiver Operating Characteristic (ROC) curve (top) and the average trajectories (bottom) of the five proteins with an Area Under the Curve (AUC) > 0.7, which were significantly different between survival and mortality in at least, four time points. The AUC is highlighted in blue. (C) ROC curve of the biomarker model built using the five significant proteins.

**Table 3:** Prediction of Unlabeled Samples for Model Evaluation Using the ROC Curve with Five Significantly Altered Proteins throughout the Disease

sample_id	probability	prediction	group	evaluation
A_10	0.91525414	SURVIVOR	SURVIVOR	right
A_22	0.73132406	NON-SURVIVOR	NON-SURVIVOR	right
A_24	0.86638253	SURVIVOR	SURVIVOR	right
A_29	0.78370859	SURVIVOR	NON-SURVIVOR	wrong
B_2	0.66794949	SURVIVOR	NON-SURVIVOR	wrong
B_27	0.52868593	SURVIVOR	SURVIVOR	right
B_31	0.76589008	NON-SURVIVOR	NON-SURVIVOR	right
B_6	0.72151331	SURVIVOR	SURVIVOR	right
F_1	0.57709965	SURVIVOR	NON-SURVIVOR	wrong
F_10	0.92672567	SURVIVOR	SURVIVOR	right
F_11	0.62715353	SURVIVOR	NON-SURVIVOR	wrong
F_12	0.80251517	SURVIVOR	SURVIVOR	right
F_2	0.63318713	NON-SURVIVOR	NON-SURVIVOR	right
F_3	0.60366949	SURVIVOR	NON-SURVIVOR	wrong
F_4	0.84980929	NON-SURVIVOR	NON-SURVIVOR	right
F_5	0.82105303	SURVIVOR	SURVIVOR	right
F_6	0.75418397	SURVIVOR	SURVIVOR	right
F_7	0.85126987	SURVIVOR	SURVIVOR	right
F_8	0.72222841	SURVIVOR	SURVIVOR	right
F_9	0.91094671	SURVIVOR	SURVIVOR	right

## Discussion

### Changes in Proteome Profiles during COVID-19

Proteomic analysis of plasma changes caused by COVID-19 over time is crucial to understanding the progression of the disease in an unbiased and comprehensive manner. In this study, we examined an extensive set of plasma proteome data obtained from 197 samples of 44 patients during six-week hospital admission. We integrated plasma processing using the S-Trap methodology with data acquisition via DIA on the mass spectrometer, providing broad coverage of the plasma proteome. Overall, statistically significant changes in the abundance of 828 proteins were observed over time. We identified two main protein groups: the first group shows a decrease in protein abundance over the weeks and is characterized by processes associated with the acute phase and inflammation. The early immune response in patients at hospital admission is typically characterized by acute activation accompanied by a complex interplay of pro- and anti-inflammatory signals.^15^ During treatment, these immune processes progressively declined over time, approaching the average levels observed across the cohort. Similar results have also been described. Geyer^12^ et al., conducting a longitudinal proteomic study with 31 patients infected with COVID-19, covering a period of 14 to 54 days identified a group of proteins regulated negatively over time, associated with the immune system, including proteins from the innate immune system and inflammation. In addition, longitudinal studies of inflammatory markers, determined by routine clinical biochemistry and immunoassays, also detected peaks levels of IL-6, CXCL10, and IL-10 after 24–72 h of hospitalization but declined thereafter before patient dispatch.^16^

On the other hand, the second group showed an increase in protein abundances over time, with enriched biological annotations related to vesicle-mediated transport, endocytosis, and antigen binding. During infection, platelets activate vesicle-mediated transport and endocytosis machinery which is associated with extracellular vesicle (EV) biogenesis and viral internalization. In the context of plasma there is increased abundance of antigen-binding proteins like immunoglobulins to mount the humoral response. Also, protein and lipid content of EVs influence the metabolic responses of different recipient cells. EVs in the recovery phase, for example, had an opposite effect on cellular processes when compared to those in the hyperinflammatory phase.^17^

Another group of coregulated proteins observed in the global correlation map was related to the insulin-like growth factor (IGF) pathway. The six insulin-like growth factor binding proteins (IGFBPs) are multifunctional proteins that modulate the access of IGF-1 and IGF-2 to their primary receptor (IGF1R), which can then regulate various functions such as cell survival, migration, and metabolism. The importance of this pathway in regulating the immune response has been previously described.^18,19^ Regarding COVID-19, studies have shown protein association from this pathway with the severity of the disease. Fan^20^ et al. investigated the association between circulating IGF-1 and mortality risk using data from the U.K. Biobank, finding that of the 1,670 COVID-19 patients analyzed, those with higher IGF-1 concentrations had a 41% lower mortality risk compared to the lowest quartile. Mester^21^ et al. also showed that higher serum levels of IGFBP-2 are associated with increased disease severity in COVID-19 patients. Obese patients with COVID-19 had lower IGFBP-2 levels and no significant differences in IGFBP-2 levels were detected between male and female COVID-19 patients; women in the control group had higher IGFBP-2 levels compared to men.^21^ In our study, IGF-binding proteins showed an upward trend over time (Figure 3B), which correlated negatively with disease progression. This means that the more acute the patient’s condition, the lower the levels of these proteins. Similar results were found by Ilias^22^ et al., when measuring IGF-1 and growth hormone (GH) levels along with other clinical parameters in 209 critical and noncritical COVID-19 patients and 39 controls. They observed that patients who did not survive COVID-19, particularly those in intensive care, had lower IGF-1 levels, suggesting a potential link between low IGF-1 levels and severe outcomes.

### Differential Response of Nonsurvivors Compared to Survivors

Statistical analysis revealed that the overall proteomes abundance did not differ between the outcome groups during the first 3 weeks of hospitalization. However, the temporal dynamics of specific protein subsets diverged markedly from the fourth week onward. The variation in the patient’s outcome has also been observed in other proteomic and clinical studies. For example, Demichev et al. evaluated a group of clinical parameters, plasma proteomes, cell counts, and enzyme activities to classify survivors from nonsurvivors, but these measures performed poorly in distinguishing between these patient groups, especially during the initial days of sampling.^5^ In our case, the comparison between deaths and survivors using the Student’s *t* test showed the complement pathway more activated in patients who died compared to those who survived. However, by the third week, blood coagulation or humoral response decreased in the deceased patients. From the fourth week onward, the profile of the deceased showed the acute phase and inflammatory responses more prominent compared to survivors. Demichev^5^ et al. observed that an increase in specific acute-phase and inflammatory proteins over time (e.g., SAA1, SAA2, CRP, ITIH3, LRG1, SERPINA1, and LBP) was associated with the risk of death from COVID-19. Additionally, Lee^23^ et al. reported 46 differentially expressed proteins in severe COVID-19 patients compared to nonsevere patients, most of these proteins were associated with the humoral response and acute-phase response. The increase of these processes in patients who did not survive after the fourth week may be more related to dysregulation of the immune response rather than a direct viral cytopathic effect.^24^

To find proteins that could help predict patient outcomes, we calculated the area under the ROC curve for proteins differentially expressed between death and discharge groups during the first 2 weeks. Five proteins (HPR, IGFALS, PF4, LPA, and COLEC11) were found to have high accuracy in distinguishing “death” patients from “discharged’ patients during the first 2 weeks (area under the ROC curve >0.72), and these proteins were also differentially expressed in at least 4 weeks according to the statistical analyses performed. Using the SVM algorithm, a model was created that could classify the patients’ outcomes (discharge or death) in the first 2 weeks of hospitalization with high efficiency (AUC of the ROC curve 0.801, (95% CI: 0.622–0.98)). To validate the predictive power of the model, 20 unlabeled samples were analyzed, considering only the samples from early time points, the model yield an overall accuracy of 75%. This indicates that the model is promising for classifying patient outcomes based on these five significant proteins and may be useful in clinical practice. However, it is important to consider other metrics and validations before applying it widely. Although the limited number of early time point samples increases the risk of overfitting, several aspects of our modeling strategy help mitigate this concern, including the use of a strict leave-one-patient-out framework, the absence of automated feature selection, and the biological rationale underlying the five selected proteins. Also, it is important to highlight that patients were randomly selected from the biobank to reduce systematic bias related to classical clinical predictors such as age, sex, and comorbidities. Consequently, the predictive performance of the biomarker panel could reflects molecular signatures linked to disease progression, suggesting that plasma proteomics provide complementary prognostic value beyond standard predictors.

These proteins had previously been related to disease severity. For example, in a study by Völlmy^25^ et al., both HPR and IGFALS were differentially expressed in nonsurvivors compared to survivors during the first 7 and 14 days, with HPR being less expressed at all time points studied. The protein IGFALS also has negative correlation with disease progression.^18^ Kimura^26^ et al. (2021) employed quantitative proteomic analysis identifying serum proteins associated with favorable or adverse outcomes in severe COVID-19 patients and identified IGFALS as a potential biomarker of adverse disease prognosis. The ROC curve AUC values for predicting the clinical prognosis of severe patients with IGFALS was 0.84 (95% CI, 0.723–0.939),^26^ a value very similar to ours for unfavorable prognosis (0.803, 95% CI, 0.66–0.904).

Platelet factor 4 (PF4) has several roles in maintaining normal physiological processes, as blood cell formation, immune response, and cell communication. Recently, it has earned significant attention due to its role in vaccine-induced immune thrombotic thrombocytopenia (VITT) following the COVID-19 vaccine.^27^ PF4 can bind to heparin, producing IgG antibodies, and contributing to heparin-induced thrombocytopenia (HIT).^28^ In a study by Liu^29^ et al., it was observed that most severe COVID-19 patients developed anti-PF4 antibodies even without the effect of COVID-19 vaccination.

Collectin 11, (COLEC11), also known as renal collectin 1 (CL-K1), is part of an important group of collectin proteins, which are associated with the recognition of microbial molecular patterns. Collectins facilitate elimination through various mechanisms of aggregation, complement activation, and opsonization.^30^ In a study investigating rare genetic variants of the complement system, it was found that the proteins COLEC11, COLEC10, and MASP1 could offer protection against severe forms of COVID-19 by mitigating the activity of the lectin and prothrombin pathways. Furthermore, the study observed enrichment of certain variants of these three genes in asymptomatic individuals over 60 years old, which was associated with a decrease in lectin pathway activity in serum and reduced expression of these proteins in lung tissue.^31^

Lipoprotein A (LPA) belongs to the group of apolipoproteins, which are functional proteins involved in lipid homeostasis in blood. LPA has pro-inflammatory, pro-thrombotic, and pro-atherogenic properties.^32^ Several studies have shown an association between LPA and the risk of thrombosis during COVID-19.^33,34^ Thrombotic activity, marked by elevated D-dimer levels, was associated with higher LPA levels in COVID-19 patients, while elevated inflammatory biomarkers like IL-6, CRP, and procalcitonin were associated with lower LPA levels.^35^ LPA levels increased during the first 3 weeks of admission in both groups (Figure 6B), which corresponds to previous results where an increase in LPA during the first 21 days of hospitalization correlated with the incidence of venous thromboembolism in infected patients.^36^ However, in our study, in the last week, LPA levels dropped drastically in patients who did not survive.

### Limitations

Despite yielding valuable insights, this study has several important limitations. The absence of preinfection baseline samples or a healthy control group prevents us from distinguishing SARS-CoV-2–specific proteomic alterations from inherent interindividual differences or previous comorbidities. Our weekly sampling over a six-week window may miss rapid, transient protein fluctuations occurring on shorter time scales, as well as long-term convalescent changes beyond the study period. Furthermore, all altered processes require functional validation experiments, and our biomarker models have not yet been tested in independent external cohorts to confirm their predictive robustness. Also, random selection of patients from biobank does not establish total independence from conventional clinical predictors such as age, sex, or comorbidities. Formal evaluation of the added predictive value of these proteomic signatures relative to established clinical variables would require dedicated multivariable modeling and comparison against clinical-only models and represents an important direction for future studies. Finally, our results primarily reflect host responses in immunologically naïve individuals, since all patients were unvaccinated. Consequently, proteomic trajectories and prognostic markers may differ in vaccinated or previously infected (hybrid-immunity) populations.

## Conclusions

In this study, we characterized longitudinal changes in the plasma proteome of hospitalized COVID-19 patients hospitalized for over 6 weeks to understand the pathophysiological response of virus infection and identify proteomic signatures predictive of patient outcomes. We observed two principal patterns of protein regulation: an early transient up-regulation of acute-phase and inflammatory mediators that gradually returned to cohort baseline, and a complementary late-onset increase in vesicle-mediated transport and antigen-binding proteins reflecting mounting humoral responses and extracellular vesicle activity. We also observed a progressive rise over time in IGFBP family members. These findings suggest a potential modulatory role of these proteins in pro-inflammatory signaling during SARS-CoV-2 infection. One of these members, IGFALS together with HPR, PF4, LPA, and COLEC11 achieved ROC AUCs >0.72 for discriminating survivors and nonsurvivors, and, when combined in an SVM classifier, yielded an AUC of 0.801 (95% CI: 0.622–98) and 75% accuracy on a validation set containing samples from early time. These results indicate that disease outcome is associated with specific proteomic patterns and supports the identification of candidate prognostic markers. Overall, this work provides insight into the temporal dynamics of the host proteomic response to COVID-19 and highlights candidate proteins for further validation and mechanistic investigation, contributing to a more refined understanding of disease pathophysiology.

## Perspectives and Significance

This study provides a comprehensive temporal map of plasma proteome changes in hospitalized COVID-19 patients, revealing dynamic host responses and early prognostic biomarkers of survival. By profiling 197 plasma samples for 6 weeks, we identified trajectories of inflammatory, coagulation, and vesicle-mediated processes, as well as the modulation of the insulin-like growth factor pathway. In addition, we identified a panel of proteins (HPR, IGFALS, PF4, LPA, and COLEC11) associated with patient outcomes during the early phase of hospitalization, suggesting their potential as candidate prognostic markers. These findings improve our understanding of COVID-19 pathophysiology and highlight the value of longitudinal proteomics in uncovering potential biomarkers. Beyond COVID-19, this framework may be applied to other infectious and inflammatory diseases where early prognostic information is essential for guiding patient management and therapeutic decision-making.

## Supplementary Material

















## Data Availability

Proteomics raw data are available in the ProteomeXchange repository with the identifier PXD069899.

## Notes

Codes of the samples collected over 6 weeks (Table S1) (XLSX)
Clinical and demographic data (Table S2) (XLSX)
Total Proteins Identified (Table S3) (XLSX)
Significant proteins identified by weekly one-sample t-tests (Table S4) (XLSX)
Significant proteins identified by weekly two-sample t test between survivors and non-survivors (Table S5) (XLSX)
PCA loadings for each week (Table S6) (XLSX)
Enrichment of differentially expressed proteins between non-survivors and survivors (Table S7) (XLSX)
Mixed effect model results (Table S8) (XLSX)
